# Contributions of Flow Cytometry to the Molecular Study of Spermatogenesis in Mammals

**DOI:** 10.3390/ijms22031151

**Published:** 2021-01-25

**Authors:** Rosana Rodríguez-Casuriaga, Adriana Geisinger

**Affiliations:** 1 Department of Molecular Biology, Instituto de Investigaciones Biológicas Clemente Estable (IIBCE), 11600 Montevideo, Uruguay; 2 Biochemistry-Molecular Biology, Facultad de Ciencias, Universidad de la República (UdelaR), 11400 Montevideo, Uruguay

**Keywords:** spermatogenesis, flow cytometry, FACS, male infertility

## Abstract

Mammalian testes are very heterogeneous organs, with a high number of different cell types. Testicular heterogeneity, together with the lack of reliable in vitro culture systems of spermatogenic cells, have been an obstacle for the characterization of the molecular bases of the unique events that take place along the different spermatogenic stages. In this context, flow cytometry has become an invaluable tool for the analysis of testicular heterogeneity, and for the purification of stage-specific spermatogenic cell populations, both for basic research and for clinical applications. In this review, we highlight the importance of flow cytometry for the advances on the knowledge of the molecular groundwork of spermatogenesis in mammals. Moreover, we provide examples of different approaches to the study of spermatogenesis that have benefited from flow cytometry, including the characterization of mutant phenotypes, transcriptomics, epigenetic and genome-wide chromatin studies, and the attempts to establish cell culture systems for research and/or clinical aims such as infertility treatment.

## 1. Biology of Spermatogenesis and Main Difficulties for Its Molecular Study

Mammalian spermatogenesis is the process of male gamete formation, a differentiation process that in normal conditions initiates at puberty and can last as long as the individual. It takes place in the testes, which bear at least seven somatic cell types, and at least 26 morphologically distinct germ cell stages [1].

**Figure 1:**
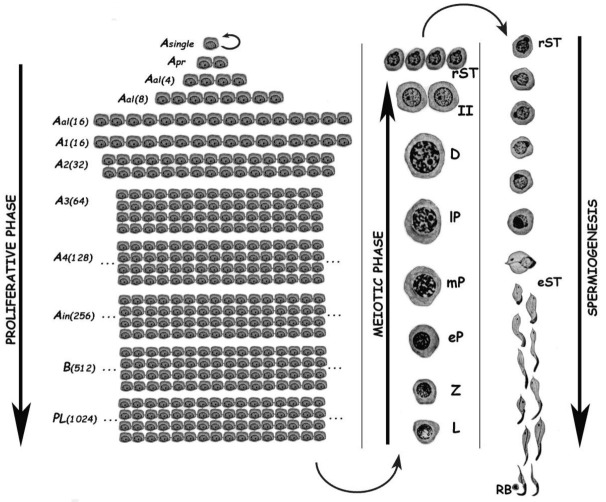
Schematic representation of the different germ cell stages along spermatogenesis of the rat. The three main phases of the spermatogenic process are indicated. *A_single_*, A single spermatogonium; *A_pr_*, A paired spermatogonia; *A_al_*, A aligned spermatogonia; A_1_–A_4_, type A spermatogonia 1–4; *A_in_*, intermediate spermatogonia; *B*, type B spermatogonia; *PL*, pre-leptotene spermatocytes; *L*, leptotene spermatocyte; *Z*, zygotene spermatocyte; *eP*, early pachytene spermatocyte; *mP*, medium pachytene spermatocyte; *lP*, late pachytene spermatocyte; *D*, diplotene spermatocyte; *II*, secondary spermatocyte; *rST*, round spermatids; *eST*, elongating spermatids; *RB*, residual body. In the proliferative phase, the numbers of spermatogonia derived from an A single are indicated in parenthesis.

Spermatogenesis consists of the successive occurrence of three main phases: (i) the proliferative phase of spermatogonia; (ii) the meiotic phase; and, (iii) the terminal differentiation phase or spermiogenesis (Figure 1). All three phases are essential for the reproductive health of the individuals, and consequently for the species survival.

The proliferative phase depends on the presence of spermatogonial stem cells (SSCs) that amplify themselves to maintain the SSC pool (self-renewal), but also give rise to progenitor spermatogonia (committed to differentiation). For instance, in the mouse SSCs are derived from gonocytes and reside in the population of *A_single_* (*A_s_*) spermatogonia. The latter initiate mitotic proliferation either to produce new *A_s_* spermatogonia by complete cytokinesis, or to give rise to chains of *A_paired_* (*A_pr_*), and *A_aligned_* (*A_al_*) spermatogonia. These represent undifferentiated spermatogonia that are connected by intercellular bridges as a consequence of incomplete cytokinesis. *A_al_* spermatogonia, as well as a few *A_pr_* spermatogonia, differentiate into *A_1_* spermatogonia without division, and then proliferate mitotically five times to sequentially form *A*_2_, *A*_3_, *A*_4_, intermediate, and type *B* spermatogonia, collectively termed differentiated spermatogonia. Afterwards, type *B* spermatogonia divide into two primary diploid spermatocytes that enter meiosis [2].

In the meiotic phase, ploidy halving is accomplished through a single round of DNA replication followed by two cellular divisions. During the first meiotic division (meiosis I) homologous chromosomes segregate, and primary spermatocytes (4C, 2n) give rise to secondary ones with 2C DNA content but already haploid (1n). Secondary spermatocytes enter meiosis II, and separation of sister chromatids takes place, generating the round spermatids (1C, 1n), which initiate spermiogenesis. Notably, the reduction in ploidy is of fundamental importance for gametogenesis in all sexually reproducing organisms, as at the time of fertilization fusion of the male and female gametes leads to the restoration of the species chromosome number. In addition to its reductive nature, meiosis is also very peculiar regarding the exchanges of genetic material that take place between homologous chromosomes during prophase I. Homologous chromosomes (i.e., of maternal and paternal origin within each pair) align, and then synapse via a highly specialized proteinaceous structure—the synaptonemal complex (SC)—that assembles during prophase I, enabling the closeness required for homologous recombination [3,4]. Due to the importance of the unique events that take place during meiotic prophase I (formation of the SCs, alignment and pairing, recombination), this has been the most extensively studied meiotic stage. As it is a very long stage, it has been divided into different substages to facilitate its study: leptotene (L), zygotene (Z), pachytene (P), diplotene (D), and diakinesis (see Figure 1). The assembly of the SC starts in L, homologous pairing takes place in Z, and recombination (crossing-over) is the hallmark of P. During D, the SCs disassemble [5]. The eventual alteration of these events often leads to spermatogenic arrest and infertility [6,7,8,9].

Spermiogenesis is the third and final phase of spermatogenesis. Along this post-meiotic stage, round spermatids go through a series of profound morphological and functional changes, giving rise to mature spermatozoa (see Figure 1). In the mouse, spermatids can be morphologically classified as steps 1–8 round spermatids, and steps 9–16 elongating ones [10]. Particularly in the chromatin, the main change in spermatids is the replacement of most histones by transition proteins first, and then by protamines, which leads sperm DNA to an extraordinary level of compaction [11,12].

In addition to germ line cells, the mammalian testis contains specialized somatic cell types that support spermatogenesis [13]. Amongst them, Sertoli cells are located inside the seminiferous tubules along with the developing germ cells, and through their intercellular tight junctions form the blood–testis barrier, which provides the isolated environment necessary for the development of spermatocytes and spermatids. Additionally, Sertoli cells provide paracrine support to all adult germ cell types [14]. Leydig cells reside in the interstitial tissue outside the seminiferous tubules, and have a fundamental steroidogenic function, providing the testosterone needed to drive spermatogenesis [15].

Mammalian spermatogenesis takes place in an asynchronous way, with a wide variety of developmental stages simultaneously present in the male gonad, and processive waves of retinoic acid (RA) propelling the asynchronous and continuous sperm production [16]. This cellular heterogeneity represents a major drawback for the identification of molecular factors, and the unveiling of molecular mechanisms underneath gamete formation. Studies seeking these goals usually require the isolation of cells from the developmental stage of interest. The lack of an effective in vitro culture system [17,18] has also hampered spermatogenic stage-specific molecular studies. The difficulties are even worse for short-lasting stages such as early meiotic prophase I stages (L and Z), since stage brevity is reflected at the histological level as cell scarceness.

## 2. Most Common Approaches to the Study of Spermatogenesis

Postnatal testis development has been studied in detail for several species, generating information on the post-partum timing of appearance of specific cell types along the first spermatogenic wave [19,20,21]. Before engaging in spermatogenic cell purification issues, it should be mentioned that many groups have made use of the first spermatogenic wave and employed whole testes from juvenile animals of different ages for downstream molecular studies [22,23,24,25,26,27,28]. Altogether, these studies have enabled correlations between the observed molecular changes, and the appearance of certain cell types. A major advantage of this approach is the use of minimally-manipulated testicular tissue as source material. However, it also has a non-minor disadvantage, since molecular analyses starting from such a complex tissue cannot precisely distinguish which cell types are responsible for the observed changes. Results interpretation is much more straightforward and unambiguous when the starting material is more homogeneous (i.e., isolated or enriched stage-specific cell populations).

Regarding testicular cell separation approaches, two main techniques—STA-PUT and centrifugal elutriation—have been classically employed. Both of them are based on the differences in size and density among the different cell types [29]. STA-PUT consists of the gravimetric decantation of cells in an albumin gradient [30,31,32] and allows the obtainment of enriched populations of P spermatocytes and round spermatids (75% and 81%, respectively [29]). Centrifugal elutriation combines the intensity of centrifugal force with liquid flow velocity [29] and can produce an enrichment of over 80% for P spermatocytes and round spermatids ([29], and our own experience). Very poor enrichment in other spermatogenic stages is obtained by both techniques.

A third common approach to analyze—and eventually isolate—spermatogenic cells is flow cytometry (FCM), which allows the distinction of a higher number of subtypes of male germ cells and at a higher purity rate than the two previously mentioned, as will be referenced in the following sections. So far, the number of papers and approaches where FCM has been used, either as an analytical or preparative tool, is huge, and it would not be possible to cover all of them. Therefore, this review intends to outline different applications where FCM has contributed to the molecular knowledge and assessment of mammalian spermatogenesis and provide illustrative examples of some of those applications.

Noteworthy, single-cell approaches have recently started to be applied to spermatogenesis analysis, as will be elaborated later in this revision.

## 3. The Spermatogenic Process from a Flow Cytometric Perspective

As mentioned above, spermatogenesis comprises several stages from spermatogonia to elongated spermatids, which present a wide range of variation in their cellular size, shape, inner complexity, chromatin structure, and DNA content. Most these parameters can be detected and measure-assigned by FCM, thus explaining why this powerful technology came to stay in this research area [33]. Table 1 summarizes some important features of the most relevant testicular cell types that are useful in FCM.

**Figure 2:**
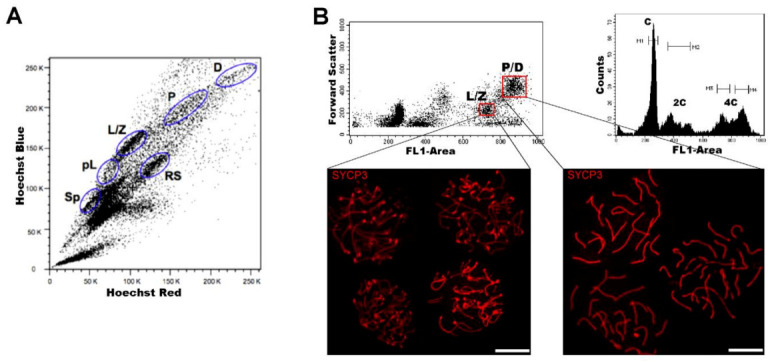
FCM analysis of mouse testicular cell suspensions employing two different DNA dyes. (**A**) Representative Hoechst 33342 (Ho342) FCM profile obtained from the analysis of adult mouse testis, based on emitted blue and red fluorescence. The various spermatogenic cell populations that can be distinguished, and are indicated, are: spermatogonia (Sp), pre-leptotene (pL), leptotene–zygotene (L/Z), pachytene (P), diplotene (D), and round spermatids (RS). This image is reproduced from reference [51] with permission of *J. Vis. Exp.* (**B**) Representative FCM profiles from the analysis of a 22-day-old mouse testicular cell suspension stained with Vybrant DyeCycle Green (VDG). A Forward Scatter vs. FL1-Area (VDG fluorescence intensity) dot plot and its corresponding histogram are shown. Histogram peaks corresponding to C, 2C and 4C cell populations, and sorting gates within the 4C population (delimited in red) in the dot plot, are indicated. Note the absence of the apparently sub-haploid peak in the histogram, as the profile is from a juvenile animal. Examples of immunolocalizations of SYCP3 (synaptonemal complex (SC) protein 3, a lateral-element–SC component) on cellular spreads from sorted cells, in order to confirm the purity of the sorted fractions, are shown below. As can be seen, cells coming from the selected gates correspond to different stages of the first meiotic prophase: L/Z, in which simple axes (L) and short stretches of SCs (Z) are present; or P/D with completely assembled (P), or disassembling (D) SCs. Bars correspond to 10 µm.

**Figure 3:**
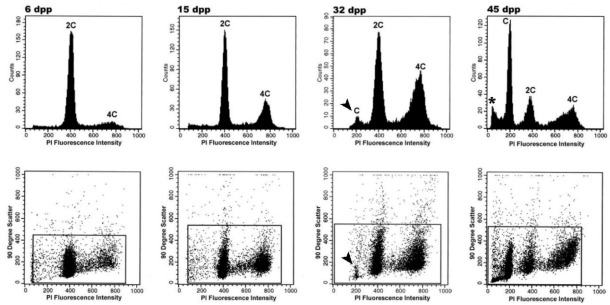
Spermatogenesis advance during postnatal testis development in guinea pig, followed through FCM. Representative profiles for selected ages indicated as days postpartum (dpp) are shown. Histograms correspond to propidium iodide (PI) fluorescence intensity, and dot plots depict 90-degree scatter (side scatter) vs. PI fluorescence intensity. Spermatogenesis progress in maturing specimens is visualized through variations in the cell populations, according to their DNA content. Note the increased frequency of 4C cells as meiosis progresses (6–32 dpp). Similarly, at 32 days after birth, the initiation of spermiogenesis is evidenced through the appearance of the first cells with C DNA content (arrowheads). Note the presence of an apparently sub-haploid peak (asterisk) at 45 dpp, corresponding to advanced elongated spermatids and sperm. This figure is reproduced from reference [21] with permission of John Wiley & Sons; permission conveyed through Copyright Clearance Center, Inc.; license number 4963790779747.

Regarding DNA content, testicular cell suspensions can be stained with a fluorescent dye and analyzed by FCM. The choice of dyes that stoichiometrically bind DNA allows the discrimination of cell populations with different ploidy based on their fluorescence intensity. In the spermatogenic context, with many cell types differing in their DNA content, this sole parameter enables a gross first simplification concerning testis heterogeneity. Three main groups of events can be distinguished: C (round spermatids, elongating spermatids), 2C (several types of G1 spermatogonia, secondary spermatocytes, testicular somatic cells), and 4C (different stages of primary spermatocytes, G2 spermatogonia) (see the histogram in Figure 2B). In adult specimens, a fourth population (elongated spermatids, sperm) can be observed, bearing an apparently sub-haploid DNA content [36] (see the histogram in Figure 3, 45 dpp). Different fluorochromes with distinct action mechanisms have been employed for the DNA content-based discrimination of testicular cell populations (for a revision, see [33]). When the aim is to classify cells for downstream molecular applications, then the vital dye Hoechst 33342 (Ho342) has been the most commonly used dye [37,38,39,40,41,42,43,44]. Ho342 is excited in the UV range and emits in blue and red; a combination of both fluorescences enables the resolution of various testicular cell populations (Figure 2A), including different meiotic sub-stages [45,46], and a side population that contains male germ stem cells [47,48]. On the other hand, we have developed an alternative sorting protocol for testicular cell populations based on the vital dye Vybrant DyeCycle Green (VDG) (Figure 2B). As VDG is excited with a blue laser (thus avoiding the potential damage to nucleic acids caused by UV light), it represents an advantage for downstream applications where nucleic acid integrity is an important issue [49,50].

Regarding cellular size, developing male germ cells exhibit a wide size range, with elongated spermatids in the lower extreme (~4–5-µm-long head), and late prophase I spermatocytes in the upper one (almost 20 µm diameter). Forward light scattering (FSC-H) allows size-based discrimination.

Furthermore, as the various testicular cell types exhibit different granularity, cellular complexity or granularity is another important aspect in aiding the distinction of stages or cell types present in highly heterogenic testicular cell suspensions, which is accomplished through side scatter parameter (SSC-H, also named 90-degree scattering).

The combination of these three parameters gives rise to multi-parametric analyses with higher information input than when considering just one, thus enabling an advance of one more step towards the distinction of different cell types and stages [33].

## 4. FCM as an Analytical Tool of Spermatogenesis

### 4.1. Testis Postnatal Development

Analyses of testicular cell suspensions employing FCM were reported for the first time more than 40 years ago [52]. These authors characterized the changes in nuclear fluorescence intensity that occurred during mouse spermatogenesis and related them to the DNA content of cells at various stages of maturation. Since then, FCM has become a widely accepted means to analyze testis developmental schedule and cell composition.

A well-recognized advantage of FCM is its high quantitative analytical power and statistical weight. Depending on the equipment, hundreds or even thousands of events per second can be analyzed. Reproductive biologists have harnessed the analytical power of FCM for multi-parametric analyses of testicular cell suspensions. They have taken advantage of the various differences between cell types and stages, which translated to FCM profiles, allow the assessment of spermatogenic advance.

Multi-parametric FCM analyses have been historically employed to study postnatal development of the male gonad in different species. Many such reports exist for diverse mammalian species, including mouse [53,54], rat [20,55], guinea pig [21], hamster [56,57], cat [58], pig [59], and several primates [60,61] including man [62,63], among others.

The results coming from some of these studies have been useful in the identification of animal models with some advantage over others. That was the case for guinea pig, in which the establishment of the first spermatogenic wave followed by FCM analyses of testicular cell suspensions from juvenile specimens at different ages after birth (Figure 3), evidenced peculiarities concerning the length of early meiotic prophase stages (L/Z). Particularly, this study identified longer L/Z stages in guinea pig compared to classical models such as mouse and rat (and hence their higher representation in the seminiferous ephitelium) [21], which enabled the purification of comparatively high numbers of L/Z aside from P spermatocytes, for molecular studies. The development of novel strategies in the recent years has opened other possibilities, as will be considered in the next sections.

### 4.2. Rapid Spermatogenic Profiling for Infertility Research and Clinical Aims

**Figure 4:**
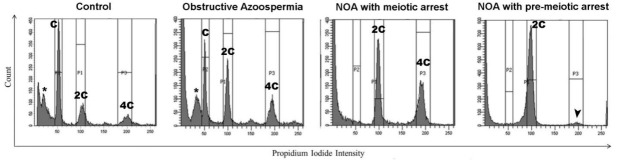
Representative FCM profiles of testicular biopsy samples from control and azoospermic patients. The testicular tissue samples were digested with collagenase and the resulting cell suspensions were fixed with 70% ethanol, stained with PI, and analyzed by FCM [64]. Obstructive azoospermia patients rendered FCM histograms undistinguishable from those of healthy control individuals, exhibiting C, 2C and 4C populations, as well as the typical apparently sub-haploid peak (asterisk) that would correspond to sperm. Non-obstructive azoospermia (NOA) patients exhibited profoundly altered FCM profiles, evidencing incomplete spermatogenesis. These analyses were also informative on the stage when the detention occurred, as NOA samples with meiotic arrest lacked the C population but presented the 2C and 4C populations, while NOA patients with pre-meiotic arrest only exhibited the 2C population. In the latter, a minimal 4C peak that would correspond to G2 stage spermatogonia could be also observed (arrowhead). The figure is reproduced from [64], under the Creative Commons Attribution License.

FCM profiling of testicular cell suspensions has not only proved useful to analyze testis postnatal development in normal individuals of different species but can also aid diagnosis in cases of human male infertility. In a recent report, FCM was employed for rapid investigation of spermatogenesis status in patients with azoospermia, as compared to healthy controls [64]. By analyzing the FCM profiles of testicular biopsy samples, the authors were able to rapidly discriminate cases of obstructive azoospermia (when the defect is caused by an obstruction for the exit of spermatozoa, for example in the vas deferens) from those of non-obstructive azoospermia (NOA; i.e., when the defect lies in the inability to produce sperm). While FCM profiles for obstructive azoospermia patients showed no evident differences with control samples, NOA patients exhibited dramatic alterations in their profiles, with disrupted spermatogenesis (Figure 4). In addition, FCM profiling in NOA patients allowed the discrimination between pre-meiotic arrest (only diploid cells present) and meiotic arrest (diploid and double-diploid cells present), helping to shed some light on the timing of the failure that eventually led to the infertile phenotype [64].

The use of FCM analysis and fluorescent dyes has been also applied in assessing sperm quality [65]. A successful conception requires fertilizing sperm with an intact haploid genome, functional competent membranes, intact acrosome, and functional mitochondria. Genetic and environmental factors may result in sperm vulnerability to damage during spermatogenesis and maturation, which in turn may cause chromatin fragmentation [65]. The two most common and reliable techniques for assessing chromatin integrity are sperm chromatin structure assay (SCSA) and Terminal deoxynucleotidyl transferase (TdT) mediated dUTP nick end labeling (TUNEL), and they are both readable through FCM.

SCSA (also termed the sperm DNA fragmentation test) renders a highly accurate measure of male reproductive health, representing the gold standard for sperm DNA fragmentation analysis [66]. It is based on acid denaturation of the chromatin followed by staining with acridine orange (AO). When DNA breaks are not present, AO intercalates in the double helix and emits green fluorescence. DNA with breaks is more susceptible to denaturation, and AO binds to single-stranded DNA with red fluorescence emission. Thus, AO bound to intact DNA is visualized as green, and to damaged DNA as red. This shift in the emitted fluorescence is analyzed either by microscope or FCM, with the latter having far greater analytical capacity. A DNA fragmentation index (DFI) can be obtained from these assays, which reflects sperm chromatin integrity and fertilizing capacity.

**Figure 5:**
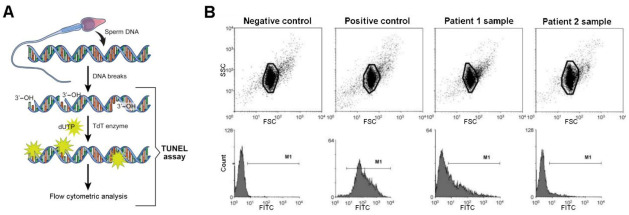
Sperm DNA integrity assessment by terminal deoxynucleotidyl transferase (TdT) mediated dUTP nick end labeling (TUNEL). (**A**) Diagram showing staining of sperm DNA for detection of DNA breaks employing the TUNEL assay. This figure is reproduced from reference [70], under the Creative Commons Attribution License. (**B**) TUNEL assay of spermatozoa analyzed by FCM. Forward light scattering (FSC) vs. SSC dot plots are represented in the top. The gates indicate the selected events for subsequent fluorescein isothiocyanate (FITC) analysis. In the bottom, frequency distribution histograms (number of events vs. FITC fluorescence intensity) of spermatozoa stained with TUNEL are shown. Negative (omitting the TdT enzyme) and positive (spermatozoa treated with DNAse I) controls were employed. The horizontal line (M1) indicates spermatozoa that are positive for the TUNEL technique, and it was adjusted arbitrarily to obtain about 1% TUNEL-positive events in the negative control. The positive control presented 98.48% TUNEL-positive sperm. Two examples of patient samples are shown. Sample from patient 1 presented a high level of TUNEL-positive cells (40.99%), while patient 2 sample exhibited a low proportion of TUNEL-positive cells (9.86%). (This figure is republished with permission of John Wiley and Sons from reference [67]; permission conveyed through Copyright Clearance Center, Inc.; license number 4977690417168).

TUNEL is another common method to detect DNA damage in sperm chromatin [67,68,69]. In apoptotic sperm, endogenous endonucleases become activated, and cut the DNA into ~200 bp fragments generating 3′-OH ends. For TUNEL detection, terminal deoxynucleotidyl transferase (TdT) is used to transfer the dUTP labeled with a fluorescent marker to terminal 3′-OH, and then analysis is performed by fluorescence microscopy or FCM (Figure 5A). Again, the high quantitative analytical power and statistical weight of FCM, makes it the method of choice whenever available. TUNEL constitutes a reliable technique that, along with basic semen analysis, can predict fertility outcome (Figure 5B) and thus guide the choice of an assisted reproductive technology procedure for infertile couples.

**Figure 6:**
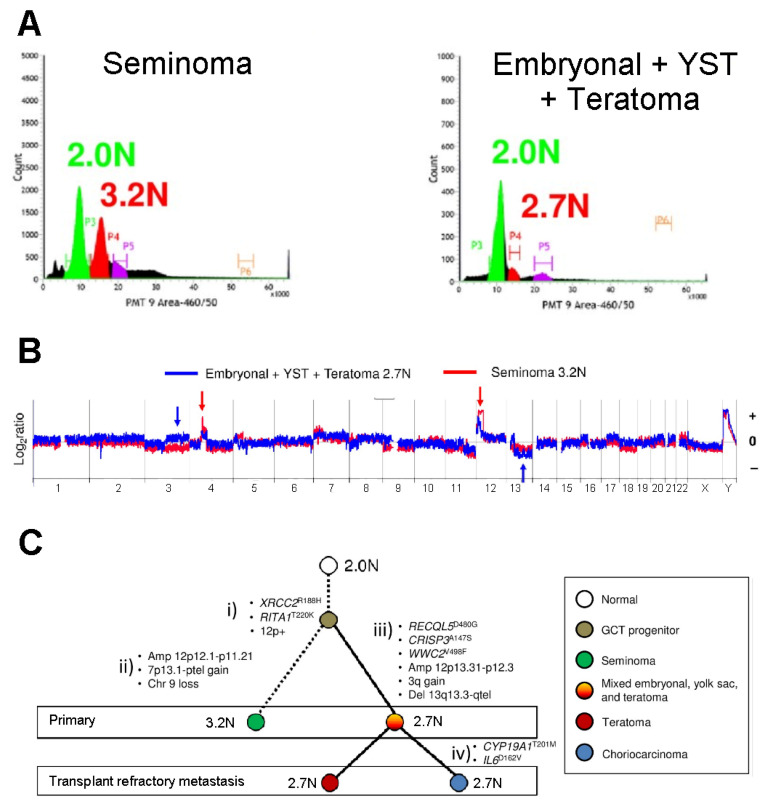
FCM profiling of testicular germ cell tumors. (**A**) DNA content FCM analysis and sorting of aneuploid (3.2N, 2.7N) and diploid (2.0N) populations from primary formalin-fixed paraffin-embedded tissues of a single patient. The histology of each biopsy is specified. Sorted cell populations were subsequently employed in whole genome copy number studies as exemplified in (**B**), and in whole exome sequencing for mutation analyses. Copy number aberrations: gains (+) and deletions (–), are indicated by arrows. (**C**) Establishment of cell lineage of metastatic testicular germ cell tumor (TGCT) through the identified genomic aberrations within primary and refractory metastatic TGCT. This figure is reproduced from reference [78], under the Creative Commons Attribution License.

FCM has also made relevant contributions regarding other medical conditions such as testicular cancer. Testicular germ cell tumors (TGCTs) arise from germ cell neoplasia in situ, originally described as atypical spermatogonia in testicular biopsies of patients that later on developed testicular cancer [71]. These tumors are classified into two main types: seminoma and non-seminomas, the latter characterized by loss of germ cell phenotype and activation of somatic differentiation. The histology of clinical TGCT samples is frequently mixed within a single tumor mass [72]. TGCTs have been shown to have low mutation rate, marked aneuploidy, and universal gain of chromosome arm 12p [73,74,75,76,77]. The aneuploid nature of TGCTs allows for DNA content-based FCM analysis and sorting (see next section) of samples of interest, which provide enriched tumor populations for downstream analyses. Barret and colleagues (2019) [78] recently described a combined approach of DNA content-based FCM, whole genome copy number analysis, and whole exome sequencing, which allowed the interrogation of the genomes of both primary and metastatic tumors, and provided a unique analysis of refractory TGCTs (Figure 6).

Another example of application of FCM testis profiling is for the characterization of genetically modified animal models. Gaysinskaya and Bortvin (2015) published a series of protocols for FCM on testis material [79]. For validation purposes, they included the application of Ho342 and propidium iodide (PI) staining to FCM analysis of testicular germ cells from spermatogenesis-defective mice, by employing a mutant line lacking the Maelstrom protein (*Mael*^−/−^), previously shown to be essential for spermatogenesis [80]. Their analysis demonstrated that the Ho342-stained cell profile of adult *Mael*^−/−^ testis was consistent with the previously reported phenotype [80], characterized as failure to complete meiotic prophase I, with lack of P and D spermatocytes and all post-meiotic stages [79].

**Figure 7:**
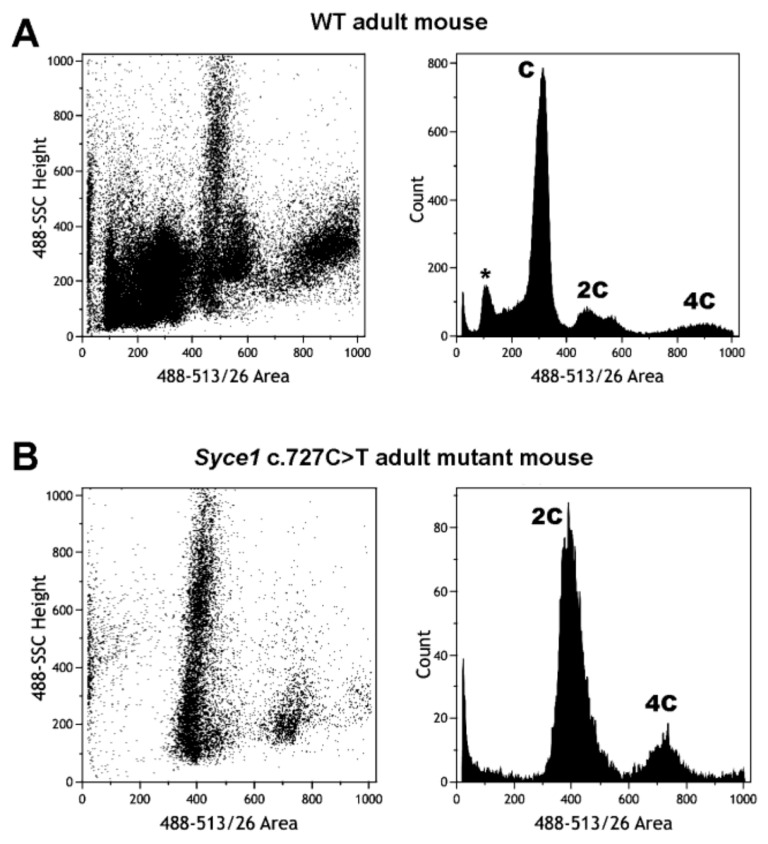
FCM analysis of VDG-stained testicular cell suspensions from adult WT and *Syce1* c.727C > T mutant mice. FCM profiles obtained for testis from WT mice (**A**) and mutant littermates (**B**) are shown. Peaks pertaining to C, 2C and 4C cell populations, as well as the apparently sub-haploid peak corresponding to sperm (asterisk), are indicated. Note the absence of cells with C DNA content and the poorly populated 4C cell population in the mutant profiles, indicating an arrest of the spermatogenic process at early stages of first meiotic prophase, in accordance with the characterization of this mutant phenotype as recently reported [81].

FCM profiling has been also included in the characterization of newly generated reproductive-disease mouse models. Idiopathic infertility (i.e., infertility of unknown cause) represents an important fraction of human infertility cases. The generation of animal models containing mutations previously found in infertile humans and suspected to be at the bases of the pathology, has proven very useful to unveil etiology and enable pathogenesis studies. A recent report from our group dealt with *CRISPR/Cas*-genome editing to generate a humanized mouse line mimicking a point mutation in *Syce1* (*Synaptonemal Complex Central Element 1*) gene [81]. Among the studies performed to characterize the humanized model mice, FCM analyses of testicular suspensions from mutant vs. wild type animals were particularly fast and revealing, evidencing a clear meiotic arrest in homozygous mutants (Figure 7). While these results were confirmed later on by light microscopy analysis of testicular cell content, FCM resulted extremely useful as a first diagnostic approach, as it is by far less time-consuming than microscopic analysis (including material preparation, sectioning, and the microscopic analysis itself). Moreover, the regular inclusion of male gonad FCM analyses in the characterization of reproductive disease models would be highly useful as a means to quantitate germ cell loss in cases of incomplete spermatogenic arrest.

## 5. FCM as a Preparative Tool in Spermatogenic Studies

The sole analysis of testicular cell suspensions by FCM has contributed in many relevant aspects to research and clinics, as exemplified in the previous sections. Whenever the available FCM equipment is also a sorter, any well-defined population in the dot plots can be chosen and classified by fluorescence activated cell sorting (FACS) for downstream studies. The strategies for the distinction and subsequent sorting of spermatogenic cell populations have been varied, ranging from multiparametric analyses that rely on differences in DNA content and light scattering, to the employment of specific antibodies against the stages of interest. The contributions of FCM sorting to the molecular understanding of spermatogenesis have been diverse and important and will be outlined in the following sections.

### 5.1. Purification of Spermatogenic Cell Populations for Transcriptomic Studies

A deeper understanding of the transcriptional program associated to the spermatogenic process has been a long-dated aim, since it has many potential applications such as diagnosis of infertility, in vitro gamete production for research and infertility treatment [82], and contraception developments [83]. Different strategies have been used to overcome the complexity of the male gonad and address transcriptomic studies along spermatogenesis, from the use of whole testes of juvenile animals at increasing ages and analysis of bulk RNA [23,25,28,83,84,85], to the isolation of testicular cell populations by STA-PUT or centrifugal elutriation either for microarray [25,86,87,88,89] or RNAseq profiling [90,91,92,93].

**Figure 8:**
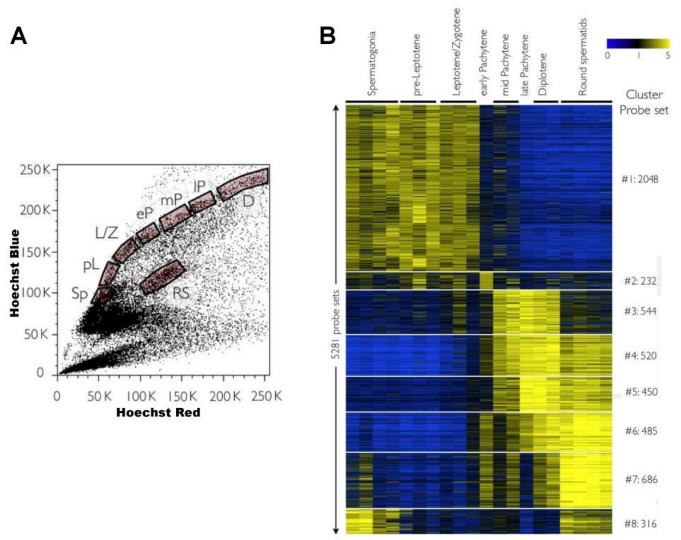
Pioneer study of massive gene expression along mouse spermatogenesis employing flow-sorted populations and microarrays. The authors [37] focused on the dynamics of gene expression along meiosis by purifying and profiling different stages of primary spermatocytes, as well as premeiotic cells and postmeiotic round spermatids. (**A**) FCM analyses and sorting were performed on Ho342-stained testicular cell suspensions based on their blue/red differential fluorescence, as shown in the dot plot. *Sp*, spermatogonia; *pL*, pre-leptotene spermatocytes; *L/Z*, leptotene/zygotene spermatocytes; *eP*, early pachytene spermatocytes; *mP*, medium pachytene spermatocytes; *lP*, late pachytene spermatocytes; *D*, diplotene spermatocytes; *RS*, round spermatids. (**B**) Heatmap representing expression levels of different gene sets along spermatogenesis. Each horizontal line corresponds to a probe set with yellow and blue indicating normalized high and low expression, respectively. The probe sets employed in this study are indicated on the right. The image is reproduced from reference [37], under the Creative Commons Attribution License.

As mentioned above, FCM allows the discrimination of a higher number of spermatogenic cell types and enables the obtainment of highly pure cell populations via FACS [20,45]; therefore, it presents important advantages for transcriptomic studies. Based on the FCM profiles of testicular cell suspensions stained with the vital dye Ho342 [45,51], Fallahi and colleagues (2010) were able to isolate by FACS an important number of spermatogenic germ cell populations from adult male mice with > 95% purity in all cases. They employed the sorted spermatogenic cell fractions in microarray chips for massive transcriptome profiling, representing a pioneer study in the combination of FCM with transcriptomic studies [37] (Figure 8). More recently, we applied our VDG-based sorting protocol [49,50] for RNA seq of four testicular cell populations, including two meiotic prophase I populations (L/Z and P/D) (see Figure 2B). The high purity of the sorted fractions (>95%), combined with RNAseq technology, enabled accurately establishment of the transcriptome fluctuations along spermatogenesis, both for coding and for long non-coding transcripts (lncRNAs) [94,95]. Moreover, the purification of the L/Z population allowed for the first time the comparison of the RNAseq-derived transcriptomes of early meiotic prophase I (in which essential events such as homologous chromosome alignment and pairing occur) and medium/late meiotic prophase cells (in which crossing over takes place) [94]. A comprehensive revision on the transcriptomics along male mouse gamete formation—and particularly of meiotic cells—is to be published elsewhere [96].

Where certain cell types cannot be solely purified based on ploidy, different strategies have been employed such as the introduction of fluorescent labels. As an example, Zimmermann et al. (2015) generated *Sox9*-*eGfp* knock-in mice by the introduction of a cassette into the 3′ UTR of the endogenous *Sox9* gene [97], which is expressed in Sertoli cells and encodes a transcription factor with a key role in testis development [98]. This approach allowed labeling and sorting by FACS, GFP-positive Sertoli cells at five different time points corresponding to key stages of spermatogenesis. These were submitted to RNAseq for the characterization of the dynamic changes of Sertoli cell-coding and noncoding transcriptomes along the first wave of spermatogenesis, which showed the evolving roles of these cells along the process [97]. In a different example, transgenic labeling in combination with FACS was used for the efficient identification and collection of spermatogonia from *Dazl* (that encodes a germ cell-specific RNA-binding protein) knockout testes and WT controls, which were submitted to RNAseq aiming at the identification of mRNAs sensitive to *Dazl* deletion. This study evidences a mechanism for DAZL–RNA binding and illustrates the role of DAZL as a master regulator of a post-transcriptional mRNA program essential for germ cell survival [99].

**Figure 9:**
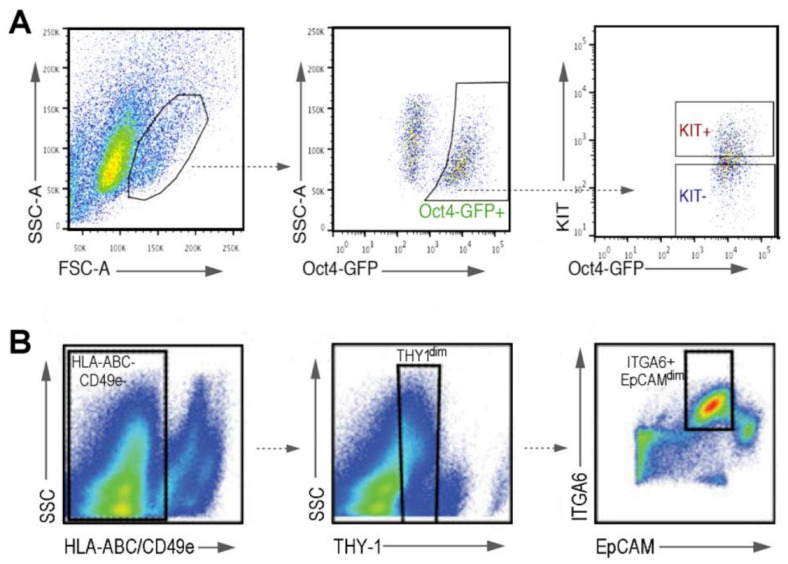
Combination of fluorescence activated cell sorting (FACS) with antibody labeling for the isolation of cell types not distinguishable in FCM profiles by sole multi-parametric analysis. Two examples are shown. (**A**) Isolation of undifferentiated germ cells from neonatal mouse testes, employing *Oct4-Gfp* transgenic mice and antibody labeling against KIT. A sorting strategy for undifferentiated germ cells (Oct4-GFP+/KIT-) by serial gating is shown. This figure is republished with permission of The Company of Biologists Ltd. from reference [113]; permission conveyed through Copyright Clearance Center, Inc.; license ID 1079949-1. (**B**) Purification of spermatogonia from adult human testes, employing FCM and antibody labeling. Sorting strategy for cells with the phenotype HLA-ABC-/CD49e-/THY1dim/ITGA6+/EpCAMdim that corresponds to undifferentiated spermatogonia, is shown. Republished with permission of Elsevier from reference [105]; permission conveyed through Copyright Clearance Center, Inc.; license number 4964010165763. In both examples (**A**,**B**), the isolated cells were subsequently employed for single-cell RNAseq (scRNAseq).

So far in this review, no specific antibody-dependent identification and sorting of cell types has been considered. This possibility does exist and, in fact, the combination of FACS with antibody labeling has been a very common strategy for the isolation of cell types that are hardly distinguishable or not distinguishable at all in the cytometric profiles, such as specific populations of spermatogonia, or certain somatic cell types. One such example is a report by Harichandan et al. (2016), who simultaneously obtained different enriched cell populations from adult human testicular biopsies by multicolor staining with a combination of lineage-specific antibodies, followed by FACS sorting. Highly enriched spermatogonia and perivascular mesenchymal stromal cells were then submitted to RNAseq, for the analysis and comparison of their transcriptomic profiles [100]. Actually, the different groups have employed diverse antibodies and strategies for sorting specific spermatogenic cell types that cannot (or are hard to) be purified based on their DNA content. A couple of examples on antibody-based sorting of spermatogonia that were then used for single-cell RNAseq (scRNAseq) are shown in the next section (see Figure 9).

FACS has been also combined with other sorting technologies for further purification. For instance, Zhu and collaborators (2016) purified 2C, 4C and C cell populations from human testicular biopsies based on ploidy by FACS, followed by subjecting the 2C population to Magnetic-Activated Cell Sorting (MACS) with anti-CD90 antibody, for the enrichment in undifferentiated spermatogonia. The sorted cell populations were subsequently used for RNAseq profiling [39].

Notably, FCM sorting has been also used as a tool to purify cell-type specific germ cell populations for proteomic analysis (e.g., [101]), although we will not address the issue here.

### 5.2. FACS Contributions in Single Cell-Based Approaches

The advent of single-cell genomics has significantly expanded the possibilities to study the highly dynamic transcriptional programs underlying sperm production. Among all recent spermatogenesis molecular reports, single-cell-based ones have gained a prominent position. Single-cell RNAseq (scRNAseq) technology enables to profile the transcriptome of thousands of single cells in a population, thus eliminating the puzzling problem of testis heterogeneity. It allows detection and characterization of the existing heterogeneity at any given phase, as well as the RNA content of rare cell populations [102,103].

The collection of high-dimensional molecular data resulting from scRNAseq is most-commonly analyzed by a pseudotime algorithm to extract latent temporal information. Pseudotime ordering measures the relative progression of each of the cells and arranges them along a continuous path representing the spermatogenic process, thus capturing the continuity of spermatogenesis.

During the last three years, there have been various reports on scRNAseq for spermatogenesis profiling, employing either human testicular samples [104,105,106,107], or mouse testes, many of which have worked with unselected cells [42,43,105,108,109,110]. As this experimental design uses the cells coming directly from a testicular cell suspension, it allows for confidence on the relative proportions of cells and prevents undesired potential consequences of selection bias such as the eventual erroneous loss of rare or transitional cell types.

In various studies, FACS has complemented single-cell approaches. For instance, some of the above-mentioned reports on scRNAseq from unselected cells have also included the analysis of certain stage-specific cells purified by FACS, for more accurate cell-type assignment or for enrichment in certain cell types [43,105,108,110]. Such is the case of Green et al. (2018), who performed FACS-mediated 1n depletion and targeted enrichment in spermatogonia, Sertoli, and interstitial cells from adult mouse, to compensate for low abundance and allow a more comprehensive evaluation of these cell types found to be relatively underrepresented in their unselected dataset [108].

Regarding methodological constraints, cell enrichments before scRNAseq profiling would have the disadvantage that altered cell proportions influence the statistics underlying pseudotime modeling. Anyway, the consequences of selection bias in the continuum pseudotime profile have proven moderate, at least in some cases. As an example, La et al. (2018) worked with selected *Plzf*-mCherry+ undifferentiated spermatogonia (*Plzf* encodes a transcriptional regulator that inhibits stem cell differentiation and is expressed in undifferentiated spermatogonia) and obtained a very similar pseudotime profile to others’ based on unselected spermatogonia [111].

The use of specific markers has been indeed a common practice when the interest was on investigating spermatogonia and addressing developmental questions such as the origin of SSCs. Therefore, many scRNAseq studies have analyzed the transcriptomes of mouse prospermatogonia and/or spermatogonia selected by means of specific markers and isolated by FACS at certain time points after birth [105,112,113,114], during the embryonic phase [115], or even from adult individuals [105] (Figure 9). Different laboratories have used a combination of transgenic fluorescent labels and antibodies, for FACS sorting (an example can be seen in Figure 9A). The purification of SSCs will be specifically discussed later on. Importantly, for detailed lists on some of the most commonly used markers for the effective FACS sorting of SSCs from the testes of different mammals, recent review articles specifically addressing this topic, and tables therein, can be consulted [116,117]). Together, single-cell analyses have identified new subsets of spermatogonia and unveiled the dynamic nature of spermatogenic initiation.

FACS—based both on ploidy and on cell type-specific antibodies—has been also employed as a parallel strategy to random cell picking (unsorted) for sample collection. For instance, Wang et al. (2018) used both approaches to ensure the capture of all the testicular cell types from human donors with normal spermatogenesis and with NOA, before scRNAseq [106]. Likewise, Jung et al. (2019), who introduced a novel model-based factor analysis method (sparse decomposition of arrays [SDA]) and applied it to the analysis of scRNAseq data from the testes of wild-type and mutant mice with gonadal defects, compared the expression profiles of cells from total testis dissociation, to those of testicular cells of known identity purified by FACS [43]. Rather than clustering groups of cells (as in the rest of the reports discussed in this section), SDA identifies components comprising groups of genes that co-vary in expression and represents a single-cell transcriptome as a sum of those components. The analysis of the expression levels of known cell type markers and comparison to the FACS-sorted cells, enabled the resolution of 32 clusters into distinct subtypes of germ cells and somatic cell populations. This analytical strategy revealed a novel level of complexity, with multiple different components even within well-recognized meiotic stages such as the P stage [43].

Thus far in this section, we have outlined reports based on the analysis of cells from specimens with unmanipulated, asynchronous spermatogenesis. In 2013, Hogarth and colleagues reported a novel synchronization method [118], which has been recently applied upstream of scRNAseq studies [40]. It consists of the administration of an inhibitor of RA synthesis (compound WIN18,446) to juvenile mice for 7 days, followed by a single dose of RA, which results in spermatogenic synchronization, with a dramatic simplification of testicular cell composition [118]. Refined versions of this synchronization protocol have been developed, which yield predictable timing of germ cell development, allowing enrichment for precise developmental stages by simple timed collections [40,119]. The latter authors also devised FACS protocols to efficiently sort the synchronized germ cells. Their 3S method (synchronize, stage, and sort) combines in vivo simplification of the cellular composition of the testis and ex vivo cell sorting, thus achieving the obtainment of germ cell subpopulations from the undifferentiated spermatogonia through late meiotic prophase with ~90% purity and high yield [108]. Compared to the unperturbed adult testis, synchronization would increase the percentage of germ cells at any given meiotic phase by a factor of at least 10 [120], allowing rapid sorting [119]. Moreover, despite the process of spermatogenesis being manipulated, synchronization would not interfere with normal germ cell biology and function, as synchronized animals have not shown overt differences in fertility or gene expression compared to control, unsynchronized testes [118,119].

Chen et al. (2018) used a combination of synchronization and transgenic labeling with *Vasa*-dTomato (which is expressed in spermatogenic cells) and *Lin*28-YFP (expressed in undifferentiated spermatogonia). This allowed the obtainment of 20 different spermatogenic cell types (including different types of spermatogonia, different stages of primary spermatocytes, secondary spermatocytes, and round spermatids at different steps of development) with over 90% purity, for scRNAseq profiling [40]. Based on their single-cell dataset, they also identified a spermatid-specific surface marker (CD63) that was very effective in distinguishing round spermatids at different stages. This represents a highly relevant finding as it raises the possibility of employing this discriminative marker to accurately isolate round spermatids at different developmental steps by FACS, for downstream molecular studies on spermatid differentiation. This finding might also have eventual implications for assisted reproduction, as round spermatids are used for intracytoplasmic injections into oocytes (ROSI, ROund Spermatid Injections), and differences between the various spermatid stages concerning their developmental potential after injection might exist. In this regard, the authors performed intracytoplasmic ROSI using FACS-enriched synchronized spermatids at different stages and obtained significant differences in the ability to promote development beyond the stage of two-cell embryo, between the different sorted spermatid populations [40]. In addition, the distinction and purification based on surface markers opens the possibility of sorting different spermatid stages from normal, asynchronous spermatogenesis.

### 5.3. FACS Contributions to Chromatin Conformation Studies

Mammalian genomes are organized into a highly dynamic chromatin structure, the regulation of which plays a critical role in biological processes involving DNA. During gamete generation, crucial DNA-based biological processes take place with associated chromatin remodeling. In meiosis, germ cells undergo programmed double strand breaks (DSBs) formation, homologous chromosome recognition, alignment, pairing, and recombination. Thus, highly dynamic chromosome movements occur along meiotic prophase I, generating peculiar configurations readily visualized at the light microscope level, such as the chromosome bouquet, i.e., a congregation of the telomeres at a nuclear envelope sector, originally described by Gelei a century ago [121,122,123]. Particularly for male gamete formation, some unique chromatin features have been identified. During the P stage, sex chromosomes undergo a process termed meiotic sex chromosome inactivation (MSCI), by which the unsynapsed regions of X and Y chromosomes are subjected to male-specific transcription silencing [124,125]. Later on, during spermiogenesis, the sequential histone replacement, first by transition proteins, and then by protamines (see above), involves dramatic chromatin remodeling [12,126,127]. In the few last years, there have been important advances in the understanding of chromatin accessibility, histone modifications, DNA methylomes, and three-dimensional (3D) chromatin structure, during mouse and/or human spermatogenesis [41,42,44,128,129,130,131,132,133,134]. Again, in many of these studies, FACS has played diverse important roles.

The contribution of epigenetic mechanisms to meiosis has been a long-relegated issue mostly due to inaccessibility to cell populations from all meiotic prophase I substages. In 2010, Getun et al. validated and refined Ho342-based FACS protocols, for the simultaneous purification of spermatogonia, pre-L, L/Z and P/D cell fractions from adult mice. The obtainment of all these cell populations (including L/Z, in which DSBs are generated), combined with micrococcal nuclease digestion and qPCR oligo-tiling assays, allowed to generate nucleosome occupancy maps, thus reporting that the cores of recombination hotspots have generally an open chromatin structure [38]. Gaysinskaya and colleagues (2014) went one-step ahead in the sorting protocols, by developing an optimized Ho342-based FACS protocol that allowed finer purifications, enabling the attainment of separate populations for L and Z spermatocytes (instead of a joined L/Z population) with significant enrichment (60–80% and 75–90%, respectively) [46]. This protocol, followed by genome-wide bisulfite sequencing, was applied to the study of DNA methylation in adult mouse spermatogonia, spermatocytes at all meiotic prophase I substages, and epididymal spermatozoa. They found that chromosomes exhibit a global transient reduction in DNA methylation in meiotic prophase, with a pronounced drop in pre-L followed by a progressive raise along the prophase stages, which suggests that key meiotic events occur in the context of dynamic changes in DNA methylation, perhaps facilitating them [41].

FACS has been also used in combination with ChIP-seq for epigenetic studies. As an example, Lesch et al. (2013) performed ChIP-seq for the H3K4me3 mark (associated with active promoters) and the H3K27me3 mark (associated with facultatively repressed promoters) and RNA-seq, on flow-sorted male and female mice germ cells at different time points during embryogenesis. This analysis, which was extended to meiotic and postmeiotic male germ cells (purified by STA-PUT), identified a set of genes that is maintained in an epigenetically poised state across sexes and across developmental stages, including haploid postmeiotic cells [135]. On the other hand, Ernst et al. (2019) combined FACS-sorted spermatocyte and spermatid cell populations from juvenile mice at different ages, with Cleavage Under Targets and Release Using Nuclease (CUT&RUN) [42], an epigenomic profiling strategy in which antibody-targeted controlled cleavage by micrococcal nuclease, releases specific protein–DNA complexes into the supernatant for paired-end DNA sequencing [136]. They characterized the epigenetic changes underlying X chromosome re-activation after MSCI and found a set of genes strongly enriched in the repressive mark H3K9me3 in spermatocytes, which then undergo extensive post-meiotic chromatin remodeling thus acquiring an active chromatin state in round spermatids [42].

**Figure 10:**
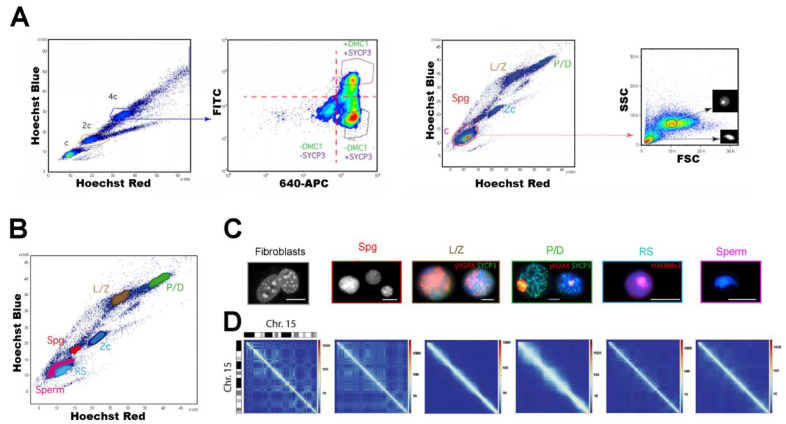
Example of employment of FACS-purified mouse spermatogenic stages in chromatin conformation studies. (**A**) Gating strategy applied to obtain L/Z (DMC1+/SYCP3+) and P/D (DMC1-/SYCP3+) stages with no cross contamination, based on re-gating the 4C population, and specific antibody labeling. The DMC1-/SYCP3- population would represent non-meiotic cells with 4C DNA content (i.e., mainly G2 phase spermatogonia). The third and fourth graphs show the gating strategy utilized to obtain separate populations of round spermatids and spermatozoa, based on re-gating the C population, and observing the FSC and SSC parameters. (**B**) Dot plot showing the purified populations that were employed in downstream chromatin conformation studies. *Spg*, spermatogonia; *L/Z*, leptotene/zygotene spermatocytes; *P/D*, pachytene/diplotene spermatocytes; *RS,* round spermatids. (**C**) Identity confirmation of the classified cells by cytomorphological analyses and/or immunolabeling. Fibroblasts and Spg have DAPI-stained DNA shown in gray. For L/Z and P/D, DAPI is shown in blue, SYCP3 in green, and γH2AX in red. In RSs, DAPI is shown in blue, and H3K9me3 (marker for the constitutive heterochromatin at centromeres–chromocenters) is red. Scale bars represent 10 µm. (**D**) Hi-C matrices for chromosome 15 at a 50-kbp resolution for the cell types analyzed. A mouse primary fibroblast cell line was employed as somatic cell control. Deep blue lines indicate non-mapped bins. The differences in the resulting Hi-C matrices evidence genome organization changes during spermatogenesis. This figure is modified from reference [44], under the Creative Commons Attribution License.

FACS-sorted testicular cell populations have been also employed in Hi-C studies (high-throughput genome-wide chromosome conformation capture sequencing), aiming at the establishment of the 3D organization of the genome in germ cells. Vara and colleagues (2019) addressed the issue by integrating Hi-C, RNAseq, and ChIPseq of CCCTC-binding factor (CTCF) and meiotic cohesins [44]. They implemented a reproducible FCM protocol to isolate enriched male mouse germ cell populations representing different stages of spermatogenesis: premeiotic spermatogonia, meiotic (L/Z, and P/D), and post-meiotic cells (round spermatids and sperm) (Figure 10). Cell suspensions were stained with Ho342, and an “in solution” immunofluorescence using primary antibodies against proteins DMC1 (a meiotic recombinase) and SYCP3 (a SC component) was performed to clearly discriminate and sort L/Z (DMC1+/SYCP3+) from P/D spermatocytes (DMC1-/SYCP3+). This sorting strategy represents an effective way to prevent cross contamination between L/Z and P/D cells (see Figure 10A), as well as to get rid of eventual contaminating G2 spermatogonia. The in-parallel application of multiple techniques on the various cell populations classified by FACS allowed the authors to find a correlation between gene expression, cohesin occupancy, and local insulation during chromatin reorganization along spermatogenesis. Among other interesting results, they observed a major structural re-organization, with dynamic changes in topological associating domains (TADs) and chromatin compartmentalization along spermatogenesis; a possible novel role for meiotic cohesins in genome organization and function during meiotic prophase I and spermiogenesis; and a differential chromatin pattern and cohesin loading in the sex chromosomes, which most likely reflects MSCI [44].

**Figure 11:**
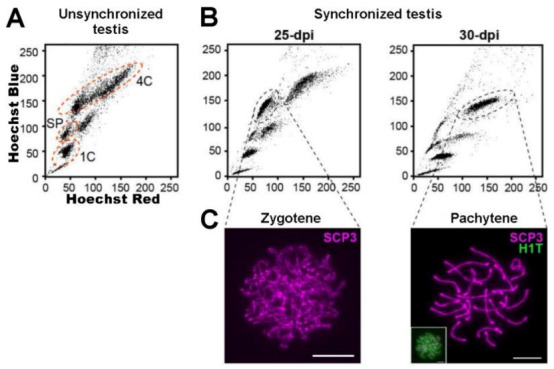
Comparison of FCM profiles for unsynchronized and synchronized mouse testes. (**A**) FCM Ho342 profile for normal, unsynchronized mouse spermatogenesis. *SP*: spermatogonia; *4C*: primary spermatocytes; *1C*: spermatids. (**B**) FCM profiles for mouse testes synchronized by the WIN18,446/RA method. Analyses performed at 25 and 30 days post-RA-injection (dpi) are shown. Note the well separated and densely populated 4C regions, compared to the unsynchronized testis. (**C**) Samples of FACS-sorted cells were employed for immunolocalizations on chromosome spreads, employing antibodies against SYCP3 and H1T (an H1 histone isoform present in P spermatocytes) for stage assessment. Scale bars correspond to 10 µm. Cells coming from the indicated regions were purified by FACS for downstream studies on chromatin organization dynamics during meiosis (Reprinted with permission from Springer Nature from reference [134]; license number: 4964911245305).

In another recent report, Patel et al. (2019) performed Hi-C in both early prophase (Z) and late prophase (late-P/D) spermatocytes isolated by FACS from mice with synchronized spermatogenesis by the WIN18,446/RA method, aiming to study chromatin organization dynamics during the recombination process [134]. By working with synchronized testes, they obtained FCM profiles with only a few well-separated and densely populated 4C regions, facilitating their classification (Figure 11). Besides providing information about the dynamic changes in TADs and chromatin compartmentalization during mammalian spermatogenesis, they observed that DSBs and crossovers show a strong bias toward the gene-dense compartment, suggesting a role for chromatin state in meiotic recombination. Concerning the X chromosome, in coincidence with the above-mentioned report and also with others [132,133], their results point to a distinct higher-order chromatin organization during MSCI [134].

### 5.4. Purification of Spermatogenic Cells for In Vitro Culture Developments

In vitro recapitulation of the spermatogenic process has been a precious goal among reproductive biologists for at least six decades. It represents a high challenge given the complexity of germ cell proliferation and differentiation, which require precise niche conditions and signals, including cellular interactions within the seminiferous ephitelium, and the presence of growth factors and hormones [18,137,138,139,140]. The availability of an effective spermatogenic in vitro system would facilitate, among others, studies on the requirements of the process in a controlled in vitro environment; research that is difficult and/or unethical to perform directly in vivo; studies on the molecular mechanisms of pathologies such as testicular cancer or male infertility; fertility restoration or preservation, by the production of haploid male germ cells from undifferentiated germ cells isolated from infertile adult patients, or from pre-pubertal cancer patients before the application of gonadotoxic treatments, respectively [17,138,139,141]. Different strategies and advances for the development of in vitro spermatogenesis have been devised in the last years and reviewed elsewhere [18,117,139,140,142] and are far beyond the scope of this revision.

As SSCs are responsible for the continuous production of spermatogonia that sustain spermatogenesis [143], the generation of in vitro culture systems to efficiently maintain and expand SSCs is fundamental for progress towards the above-mentioned goals [117,144]. The first step for the development of an SSCs culture system is the isolation of these cells, which is challenging as for instance in mouse testicles they are extremely scarce, representing only ~0.03% of total germ cells [145]. Since isolation of SSCs requires their distinction from other spermatogonial differentiation stages, both MACS and FACS have been employed in combination with specific biochemical markers for their sorting and culture. MACS can isolate highly purified cells from a complex cell mixture, based on cell surface antigen specificity. However, it cannot differentiate some subpopulations with distinct biological characteristics. In this regard, FACS presents important advantages for the obtainment of high purity SSCs, as it provides morphological data of cells, allows the simultaneous detection of multiple surface markers, and is informative on eventual quantitative differences of biochemical markers between cellular subpopulations (e.g., the distinction of Thy1^dim^ and Thy1^bright^ subpopulations within Thy-1^+^ populations, in human testicular cells) [146]. It is generally agreed that satisfactory results for FACS sorting of SSCs requires multiple markers. The advances in the molecular knowledge of testicular cells have allowed the identification of different molecular markers, which in turn has generated new tools for FACS purification. Accordingly, as above mentioned, recent revisions on markers available to distinguish and sort SSCs in different species have been published [116,117].

Regenerative capacity is the feature that better defines stem cells, and research on this aspect requires functional assays. Transplantation from mice donor testes into the testes of recipient mice has proven useful in assessing the regenerative capacity of different subsets of SSCs [117,138,141,145]. Again, FCM based on specific molecular markers has been a highly valuable tool in the discrimination between different stem cell subsets [147], to identify those with regenerative capacity that will lead to spermatogenic colonies within recipient testes.

As the field of in vitro spermatogenesis is a highly prolific research area with enormous clinical implications, so far the number of published reports is huge. Here we have selected only a few examples, to illustrate the wide variety of contributions of FCM to the subject.

In adult mice, it has been established that less than 10% of A_s_ spermatogonia have transplantation capability [148]. More recently, some have concluded that transplant activity is almost exclusively contained within a fraction of A_s_ marked by ID4 (a transcriptional repressor with a critical role in the balance regulation between self-renewal and differentiation of SSCs) expression, thus supporting a revised A_s_ model in which stem cell activity is limited to a subset of A_s_ [149,150]. Law et al. (2019) analyzed the kinetics and mechanisms underneath the appearance, during neonatal life, of the SSC foundational pool derived from prospermatogonial precursors in mouse, by employing a multi-transgenic reporter mouse model, FACS classification, scRNAseq, and transplantation analyses to define the SSC trajectory from prospermatogonia. Their results suggest that SSC fate is pre-programmed within a subset of fetal prospermatogonia, before the establishment of the foundational pool during early neonatal development [151].

An alternative to the revised A_s_ model was proposed by Nakagawa and colleagues a decade ago, introducing the existence of multiple and reversible paths from stem cells to differentiation [152]. This model conceived stem cell potential as a dynamic property shared by most undifferentiated type A spermatogonia (A_undiff_) and proposed that gene expression would dictate fate tendency. Recent evidence supporting this model has started to emerge. La et al. (2018), by using the *Plzf* reporter system for A_undiff_ FACS purification from mouse, uncovered an unappreciated population within the self-renewing A_undiff_ fraction marked by expression of embryonic patterning genes and homeodomain transcription factor PDX1, with potent stem cell capacity [111]. Interestingly, upon transplantation and culture, they demonstrated the existence of dynamic cell states for A_undiff,_ which interconvert between PDX1^+^ and PDX1^−^ states [111].

Velte et al. (2019) addressed the issue of how spermatogonial response to RA is regulated in mouse at the molecular level, and the importance of the microenvironment, by using in-parallel in vivo and in vitro approaches [153]. To obtain spermatogonia, they employed *Id4-eGfp* mice and isolated EGFP^+^ spermatogonia by FACS, to discriminate spermatogonia differing in their regenerative capacity (ID4-EGFP^bright^ spermatogonia are highly enriched in SSCs, while ID4-EGFP^dim^ and ID4-EGFP^−^ have no regenerative capacity and correspond to progenitor and differentiating spermatogonia, respectively [149,154]). By examining the intrinsic RA responsiveness of isolated spermatogonia maintained in vitro without somatic cells, they observed similar responsiveness in vivo and in vitro, thus indicating that differential RA responsiveness is an intrinsic feature of developing male germ cells, with minimal dependence on an in vivo microenvironment [153].

Others have addressed studies on human SSCs. As an example, Sohni and collaborators (2019), by employing human adult testicular biopsies and neonatal testes for scRNAseq, were able to detect four different clusters of undifferentiated spermatogonia. One of them exhibited the characteristics of highly enriched SSCs, for which two molecular markers were identified (LPPR3 and TSPAN33) and used in FACS purifications and RT-qPCR analyses. They also identified genes expressed by testicular somatic cells, including those encoding cell–cell signaling factors, which may prove useful in developing cocktails with factors to favor the expansion of human SSCs in vitro, for eventual in vivo clinical applications [155].

**Figure 12:**
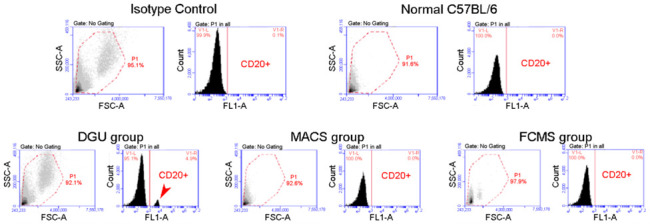
Leukemia incidence assessment after transplantation of SSC to mice with azoospermia. The authors [156] established a B cell acute lymphocytic testicular leukemia (BALL) model in mice by injection of human BALL cells. Three SSC purification groups were compared, consisting of SSCs that had been isolated and purified from the BALL model mice through either: density gradient centrifugation (DGU), flow cytometry sorting (FCMS), or immunomagnetic bead-based (MACS) sorting. In the DGU group, a large number of BALL cells (CD20+) could be detected in the blood of recipient busulfan-treated azoospermic mice 2–3 weeks after transplantation (arrowhead). Quite differently, transplantation of SSCs purified by FCMS or MACS successfully restored spermatogenesis with no incidence of leukemia after 16 weeks of observation (no BALL cells were detected in the blood of recipient mice by FCM), indicating that these two methods of purifying SSCs from the testicular tissue of the testicular leukemia mouse model could be safely applied to the SSC transplantation technology, without concomitant tumor implantation. This figure is reproduced from reference [156], under the Creative Commons Attribution License.

Despite efficient SSC purification and transplantation being a promising option for male fertility preservation in pre-pubertal cancer patients, the safety of such application is controversial, given the risk of introducing malignant cells along with the SSCs. This issue has been addressed by different laboratories. For instance, Tian et al. (2019) compared the relative safety of three different purification methods—Percoll density gradient, MACS and FACS—for application of SSC transplantation, in testes of leukemia model mice. Interestingly, after 16 weeks, no malignant cells were found in mice transplanted with MACS- or FACS-purified SSCs, which suggests that these methods could be safely applied without concomitant tumor implantation [156] (Figure 12).

Finally, we want to acknowledge the important role FCM has also played during the establishment of culture systems, for the analysis and quantitation of the SSCs in culture at different time points employing specific markers (e.g., [157]).

## 6. Conclusions and Perspectives

Being such a heterogeneous tissue, mammalian testis has represented a challenge in the attempt to unravel the molecular bases of spermatogenesis. This review was intended to illustrate the many and varied contributions of FCM to the field. However, we must recognize that due to the vastness of applications of FCM in this area, we have mainly focused on the spermatogenic process itself, leaving aside other applications such as those involving the sorting of spermatozoa (which would merit a full review on their own).

Multiparametric analysis of testicular cell suspensions enables the distinction of various cell types with no need of specific antibodies. Besides identification, FCM allows the sorting of specific testicular cell populations at high purity levels for molecular studies, which is essential for the obtainment of reliable results. For those stages that do not generate a distinguishable unlabeled population in the dot plots, there is always the possibility of using specific labels. In these cases, the identification of molecules that are specific for certain cell types or stages is obviously a pre-requisite. Innovative strategies such as spermatogenesis synchronization coupled to FACS open a whole range of possibilities, by allowing the increase in the testicular representation of the different spermatogenic cell types, or the obtainment of previously non-purifiable ones, and the characterization of the molecular signatures of those cell types. Moreover, transcriptomic techniques including single-cell transcriptomics, are identifying new stage-specific markers. In turn, this feeds the resources for more refined FCM purifications, and for the purification of an increasingly higher number of testicular cell types, thus indicating that there is still a lot of ground for potential complementation between FCM and parallel approaches.

On the other hand, modern approaches such as single-cell transcriptomics and 3D chromatin-conformation studies, incorporated into the study of spermatogenesis, are generating relevant information that is dramatically changing our perception of the whole process. FCM has revealed an extremely important complementary tool in many of these studies.

Furthermore, reproductive biologists have an enormous interest in dissecting the molecular mechanisms that drive the spermatogenic process in healthy and affected individuals, as well as achieving the long-sought goal of in vitro spermatogenesis, which has obvious important implications for clinical andrologists. Again, there is a huge potential complementation between basic research scientists and specialized clinicians. We foresee the FCM has a lot more to contribute in this area of knowledge.

## Figures and Tables

**Table 1:** Principal characteristics of testicular cells from adult rodents.

	Approximate Cellular Size (µm)	Cellular/Nuclear Shape	Inner Complexity	Chromatine Structure	DNA Content
Type A spermatogonia	12–14	Ovoid nuclei	Very low	Homogenous euchromatin	2C-4C *
Type B spermatogonia	8–10	Round nuclei	Low	Heterochromatin along nuclear periphery	2C-4C *
PreLeptotene spermatocytes	7.6–8.2	Round nuclei	Low	Heterochromatin along nuclear periphery	2C-4C *
Leptotene spermatocytes	8–10	Round nuclei	Low	Condensed chromosomes forming thin filaments	4C
Zygotene spermatocytes	10–12	Round nuclei	Medium	Clumps of dense chromatin, mainly at the nuclear periphery	4C
Pachytene spermatocytes	12–18	Large round nuclei; thin rim of cytoplasm; cell volume increases along the stage	High	Abundant clumps of dense chromatin; shorter and thicker chromosomes	4C
Round spermatids	10	Round cells with round nuclei	Very low	Homogenous euchromatin; densely stained chromocenter in the middle	1C
Elongating/elongated spermatids	4–8	Sickle-shaped nuclei	Low-Medium	Increasingly compacted chromatin	1C
Leydig cells	10–12	Polyhedral cells with eccentrically located ovoid nuclei; abundant citoplasmic lipid droplets.	Very high	Prominent nucleoli; abundant peripheral heterochromatin	2C
Sertoli cells	Height (from basal to apical surface): 90; Base: 30	Columnar, Irregular, with apical and lateral invaginations; oval-shaped nuclei	High	Dark nucleolus; central condensation area	2C

Information was extracted from references [34,35], and our own experience. * 4C DNA content in these cells corresponds to the G2 cell cycle phase. Inner complexity level assignment was performed based mainly on observation of flow cytometry (FCM) side scatter parameter.

## Data Availability

Data is contained within the article.
